# Inflammation

**Published:** 1879-09

**Authors:** Wm. H. Atkinson

**Affiliations:** New York.


AMERICAN JOURNAL

OF

DENTAL SCIENCE.

Vol. XIII. THIRD SERIES—SEPTEMBER, 1879. No. 5.

### ARTICLE I.

### *Inflammation.*

BY WM. H. ATKINSON, M. D., D. D. S., NEW YORK.

Read before the New York Dental Association.

The gradual growth to higher and better interpretations of the phenomena of inflammation, as displayed in the works of John Hunter, Virchow, Remak, Billroth, C. O. Weber, Von Recklinghausen, Conheim and Stricker, not to mention many others, are proofs of the incompleteness of the satisfaction with which each has looked upon the labors of his predecessors and co-workers, as well as his own efforts.

Till this day it is impossible for the best observers to designate the point at which physiological activity becomes a pathological mode of its presentment. Whether some of the normal operations attendant upon the ripening of ova and spermatozoids are not properly classifiable as inflammatory activities is, at least, an entertainable proposition.

Histological research has shown the "corpus luteum" to be composed of scar tissue, of a like character with scars resulting from traumatic and inflammatory lesions. Excess of blood plasm in any locality is liable to be converted into embryonal corpuscles or mucous globules, as the indifferent basis from which normal and abnormal products arise, according as the environment tends to lead to one or the other. Simple hyperplasia tends to increase of the normal tissues. When the blood tracts are obstructed, and the press of the currents great, stasis, exudation and retrogressive molecular changes take charge of the exuded blood plasm, and convert it into the products of the inflammatory process which this retrogressive metamorphosis constitutes.

Late histological discoveries strongly indicate that nerve currents and blood currents are necessary conditions to allow inflammation of any territory. All those territories, therefore, that are not supplied with blood circulation and nerve currents are, if unaided, incapable of repairing their wounds when traumatically induced. When the neural and vascular currents are coetaneous and unobstructed, it is doubtful if any mere acceleration could result in the inflammatory process; but when the heart's action is too great to exactly balance the tonicity of the capillaries supplied by the nerve currents, obstructions are readily induced in the tortuous channels of the capillary system. To prevent the inevitable advent of inflammation under such circumstances, the obstructions must be removed, and the simultaneousness of nerve currents and blood currents be regained. The simplest method of accomplishing this desirable end will be to call the part into vigorous exercise by the voluntary or involuntary effort of the subject, or by massage or the manipulation of another. The only reason why all inflammations might not thus be successfully treated depends upon our inability to cognize the departure from health at this early stage, and to get at the locality.

Swelling, redness, heat and pain have universally been grouped together as the definition of the inflammatory process. No one has put on record even an approach to legitimate, clear and satisfactory statement of the precise difference between normal nutrient function, and its derangement called inflammation.

Let us examine the process of conversion of pabulum (protoplasm) into the elements of tissue, and learn if we may that pleasure and pain, health and disease, are qualities or modes, and quantities or measure of functional activities that vanish into each other by disturbing and restoring the balances of regular molecular changes. To comprehend these, we must cognize the existence of atoms of which the molecules are composed ; and to understand the manner of the coming together of the atoms to construct the molecule, we must take into consideration the static and dynamic aspects of the atoms, no less than the awakening and engagement of the definite measures of energy that effect the combinations. Why? And how? the molecules move are the two questions the replies to which shall reveal to us the complete explanation of all functional movements, and declare how they are produced and maintained or destroyed in functioning bodies.

*Pain* is the result of obstructed sensory nerve current; *swelling* supervenes upon obstructed vascular current; *heat* is resultant upon obstructions in the neural and vascular channels, by which the mechanical movements of the masses of nerve blood and vascular blood (through impact against the points of obstruction) become changed into molecular movement, which we cognize as *heat*. These three, viz: pain, swelling and heat are the only essential factors or necessary concomitants in the process of inflammation. The redness that has heretofore been made an equal factor is a mere incident, as is proved by some swellings being white.

What are we to understand by quality or mode, and quantity or measure of function? And how are we to differentiate these predominant aspects of the complex presence we denominate function of mind and function of body? The function of mind consists of seriated arrestations and releases of the tensions of consciousness, or the power of perception. The functions of body are also seriated successions of arrest and release of tensions of combinations and separations of molecular and atomic bodies, through which the energy operates to produce molecular, granular, corpuscular, tissual, organic and systemic forms of function in their various manifestations.

Late works on chemistry assert that heat is given out or produced by combinations of atoms, and is taken up or quenched by separations of atoms. Works on physics tell us that "heat is a mode of motion," and that "mechanical motion, upon being arrested, is converted into molecular motion, which is heat." How then are we to account for the heat set free upon the admixture of sulphuric acid and water? How does the friction produce the heat so largely by the mere rushing of the molecules of water among the molecules of acid?

The partially engaged, unsatisfied or sleeping bonds of affinity belonging to the atoms in the molecules of water and acid, are in a state to be easily aroused into active motion by any impact of current within the sphere of influence, be it direct or oblique, simple or manifold, in its advent. We understand the diffusion of water molecules and acid molecules to result from the desire to attain equidistance from each other in the mass of fluid; and the rush to acquire that tension affects the awakening of the sleeping bonds of energy which, being set in motion, manifest the intense measure of heat always attending this experiment.

The difference between this process of disengagement of heat, and that form attendant upon the inflammatory process in animal bodies, consists in the greater complications of engagements and disengagements of bonds of affinity in the molecular mass in which these changes occur, and the greater freedom of the fluid in the vessel of the chemist, as compared to the confinement of molecular mass in the tissual interspaces of animal organisms. There is also a larger number of elements entering into the molecular mass, which is the basis of all animal forms of metamorphosis, than in the mass composing the vegetable or mineral examples of molecular aggregations. Hence, the increased opportunity for the display of greater varieties of molecular changes that split up and minify the thermal and other currents possible to this limited territory.

There is such a thing as having the receptacle of molecular mass so packed with molecules as to preclude any motion among them. This has been proven by a stoppage of fermentative action by the pressure of a given number of atmospheres. Take the example of a fermentable substance placed in an air-tight transparent vessel; exhaust the air by the air-pump, and then inject pure oxygen into the receptacle containing a quantity of yeast taken from the tub fresh and active. At first bubbles of carbon dioxyd (carbonic acid gas) will break and rapidly disperse into the vacuum above the yeast, there being no free oxygen present to support the fermentation, molecular change ceases to appear. Now, admit pure oxygen in a small quantity, and the fermentation is again set up with increasing activity in the ratio of the quantity of oxygen supplied, up to a given measure. So soon as the tension or pressure of the incoming current of oxygen shall interfere with the freedom of the oxygen molecules in the space above the yeast, the activity of the fermentative change gradually lessens by degrees, and finally ceases altogether. When the compression of the oxygen is great enough to arrest the motion of the oxygen molecules, they assume a static condition, preventing further changes between the oxygen and the hydro-carbons of the yeast. The yeast in the vessel is now in the condition of a "pop-bottle," or a bottle of "mineral water," fully charged with carbon dioxyd. All that is requisite to re-establish the fermentative activity is to take off the pressure from the gas above the yeast in the vessel, by giving it vent, so that the molecules of oxygen may be free to move among each other again, and then the attraction between the oxygen and the carbo-hydrates of the yeast mass is at once set vigorously at work generating molecules of alcohol and carbon dioxyd out of the already existing albumen, glucose, or diastase in it.

The alternate combination and separation of atoms to construct the varieties of molecules in the mineral, vegetable and animal primates of bodies is repeated and amplified, magnified and minified by the necessities of the types of the individual presence in the various crystals, cells and corpuscles that are the characteristics of the three kingdoms known to exist as planetary presentments. A serial order of appearance and disappearance, or coming from without and passing beyond our sensuous perception, pertains to the origination, growth and decline of every example of individual body or class of bodies, be they mineral, vegetable or animal. The chaotification of these gives the foundation from which all human functional capabilities originate; so, to understand all the activities possible to human bodies, we must become acquainted with the activities of the whole universe, of which man is the epitome.

"The whole is equal to all its parts" is an aphorism in the study of inflammation as true as it is in mathematics, astronomy and physics.

The combustion of hydrogen and carbon with oxygen is well known to produce *heat.* Friction also produces heat to the degree of ignition of combustible substances. Are we not then justified in assuming that the heat of fever and inflammation is also the product of combustion and friction? And does not this indicate that whatever prevents or arrests combustion will also arrest fever and the inflammation upon which it depends? The difference between fever and abscess is the difference between the combustion in a charcoal-pit and in an open fire. The first extracts the hydrogen from the combustible substance and leaves the particles of carbon in their undisturbed position in the charcoal or the coke; the second oxydizes carbon and hydrogen, converting them into carbon dioxyd and water, both of which conspire to change the mineral constituents, the wood or coal, into the salts of which their ashes are composed.

If we desire to understand just what fever and inflammation are, we must comprehend the process of combustion.

Combustion is the undoing of the work of the sun which constructs vegetable and animal tissues. How does this occur? Simply by the influx of an energy that enables the oxygen to disrupt the bonds of affinity of the atoms which "clamps" them together in the molecules: and the formation of other molecules by the use of these "clamps" in a different order of engagement of bonds. * * * Multivalent atoms are those which have many bonds engaged. These are placed in the "centre," or "skeleton," or "nucleus," of the molecule, around which the atoms, having few (2 or 3) bonds engaged, are distributed in a variety of ways This variation of the "skeleton" and "flesh" of the molecule is the measure of the differentiation among proximate principles. Slow oxydation of moist organic substances, especially those containing nitrogen, gradually consumes without sensible elevation of temperature. This has been called "eremacausis" (or burning by degrees.) Fermentation and putrefactien are both examples of oxydation also, but with sensible elevation of temperature. The first is distinguished by giving off no offensive gases, and by producing useful compounds; the second is characterized by the exhalation of offensive and deleterious gases. * * * By this play of affinities under the direction of the demands of the types of all forms of molecules composing human protoplasm, we have the elaboration of all the proximate principles belonging to the tissues of the human body, embryonal and adult, of a healthy or diseased character. Just how it is that the changes of position of molecules effect difference in the proximate principles of protagon, neurine, myeline, mucine, olein, margarin, cholestrine, cerebrin, lecithin, and a host of others (isolable, real and hypothetical bodies, nevertheless veritable radicals of the deciduous and permanent tissues of the body) is not just yet demonstrable without great labor with expensive apparatus.

But we do know—and can demonstrate—that tensile strength belongs to fluids as well as solids (if not to gases also.) Bubbles on pure water prove the tensile strength of $H_2O$ to be a fact, which is more easily displayed by adding soap or other viscous substance to the water, after which bubbles of larger size and greater strength may be formed, which retain their shape long enough to permit elaborate inspection. Another proof of the tensile force of water may be shown by pouring it from a perforated vessel on a smooth pool, when many spherules will be seen to roll over the face of the placid sheet for some time before melting into its body. It is said a stratum of air intervenes between the drops and the pool. But what enables each drop to retain its sphericity, if it be not the tensile strength of the water?

The rushing of the molecules of protoplasm, under the stress of the imflammatory process, has many degrees of rapidity; hence, the variety of the so-called products of this process. It may be affirmed that the first degree results in *Pus*—a simple, inoffensive body of dead blood, fluid and corpuscular. The second in *Sanies*—an offensive, decomposing mass of dead blood, evolving sulphuretted hydrogen. The third is *Ichor*—a yet further disintegration of blood, mixed with dissolved and rotten tissues, soft and hard. All that follows in this disgusting recapitulation (of demoniac possession) is the complete subversion of physiological dominion by chemical control of the elements of flesh and bone. * * *

How do we subdue fire? First, by cutting off the supply of oxygen to the fuel. Second, by quenching the flames. How is this last best accomplished? Not by water, unless it be sufficiently abundant to flood the whole body of the combustible on fire, but by enveloping the whole mass in a sheet of carbon dioxyd, or any fluid or vapor having no affinity for oxygen, or into which oxygen does not enter as a constituent. Where the fire is well kindled in an obscure building or part of a structure, it may be wise to isolate that locality, and allow the fire to burn out. Just so in local inflammations in territories that can be cut off, preventing contamination of the whole system, it will be wise to ligate or compress the channels of neural and blood supply, until, by thus establishing physiological rest, the morbific form of metamorphosis is arrested. Afterward, normal nutrition may be depended upon to spontaneously take charge of the territory upon the re-entrance of nerve and blood supply.

Paronychia ("run-around,") which is an acute inflammation, has often been aborted by persistently plunging the finger into lye of wood ashes, at the boiling-point, dipping it in and out at intervals of a few seconds, until the nerve currents and blood currents were so controlled as to discontinue the pain and swelling. Whenever this method is vigorously and persistently pursued a rapid cure is attained. Even that terribly painful periosteal inflammation called felon may be aborted or cut short by tightly bandaging the finger or thumb. To be sure, some pain may follow this method, but it is better to pursue this course, even though no anæsthetic be used to bridge the period, rather than to let it run its own course to full suppuration. The safest anæsthetic to use in such cases is ice, or ice and salt, to produce numbness of the part until the retrogressive metamorphosis may be controlled. Evaporating lotions of various substances are worthy of trial; one of the best of which is common ether (falsely called sulphuric ether.) Rhigoline is also efficient. * * *

Depletion by diaphoresis, catharsis, or phlebotomy, coupled with nauseants pushed to the accomplishment of the purpose (viz.: arrest of inflammation,) are methods within the reach of all, and absolutely harmless in the hands of ordinarily intelligent patients and practitioners.

Bandaging answers a valuable purpose in our efforts to produce physiological rest of inflamed territories. Strips of sheet rubber judiciously applied, are excellent means to drive back the excess of blood in the extremities. In cases of bruises, or loss of tone of the capillaries from any privation of nerve currents, inducing suggillation, such as the petechiæ (or flea-bite,) of low forms of fever, the application of a succession of layers of contractile collodion is an admirable means of removing the blood from these extravasated spots and from the congested capillaries. Eruptions of all sorts that appear as pimples, if left to themselves, may be successfully treated by the use of collodion applied in the manner indicated, and faithfully renewed whenever it cracks, until the danger to the face, neck and exposed portions of the skin is past.

With these principles and remedies and methods of applying them, coupled with a little common sense and entire control of the patient, no one is justified in allowing inflammations to run their natural course, as is the almost universal practice among general and special surgeons.

---

